# A Mutation in Cathepsin C Gene Causing Papillon-Lefèvre Syndrome in a Saudi Patient: A Case Report

**DOI:** 10.7759/cureus.6546

**Published:** 2020-01-02

**Authors:** Aiman Shawli, Yazan Almaghrabi, Abdullah S AlQuhaibi, Yousef Alghamdi, Abdulbari M Aboud

**Affiliations:** 1 Pediatrics, King Abdulaziz Medical City, Ministry of National Guard Health Affairs, Jeddah, SAU

**Keywords:** palmoplantar keratoderma with periodontitis, cathepsin c (ctsc), (c.899g>a p.(gly300asp)), papillon–lefèvre syndrome, papillon–lefèvre syndrome (pls), palmoplantar keratoderma

## Abstract

Papillon-Lefèvre syndrome (PLS) is a rare genetic disease that causes dermatological and dental symptoms that usually start from early age. Dermatological findings include hyperkeratoderma over the palms and soles that are usually thought of as persistent psoriasis at first. Dental findings include severe caries in the teeth that lead to premature dental loss. We present a case of an otherwise healthy seven-year-old child with classical presentation of PLS with both dermatological and dental findings. He first presented to the dermatology clinic when he was five years old brought by his parents complaining of dry scaly patches on the palm of the hands and soles of the feet. On further history it was found that he is a child of first-degree consanguinity, and he had these patches since he was four months old. On examination, he was found to have an erythematous hyperkeratotic skin plaques and papules with scales over the planter and palmar aspect of both hands with similar lesions observed on both feet, legs, scalp, and ears with nail pitting. The diagnosis of PLS was confirmed by whole-exome sequencing (WES) and the patient was started on acitretin capsules and started to show improvement.

## Introduction

Papillon-Lefèvre syndrome (PLS), also known as palmoplantar keratoderma with periodontitis, was first described by French physicians Papillon and Paul Lefèvre back in 1924 [1]. It is characterized by hyperkeratoderma over the palms and soles (palmoplantar hyperkeratosis) and generalized aggressive periodontitis leading to premature loss of primary or secondary teeth [2]. The onset of the symptoms starts early during childhood, mostly within the first five years of life. PLS is an autosomal recessive genetic disorder that is caused by a mutation in the gene that encodes cathepsin C (CTSC) [1]. PLS is an extremely rare disease with an estimated prevalence of one to four cases per million people, and it has no sexual or racial predominance [2-3]. Parental consanguinity seems to increase the risk of PLS, with 20%-40% of the cases reported being the offspring of consanguineous parents [4]. It is known that consanguineous marriages may lead to an increased expression of autosomal recessive disorders. The first case of PLS described by Papillon and Lefèvre was of two siblings of first-cousin married parents [1]. In this report, we describe the presentation of a child who was diagnosed with PLS by whole-exome sequencing (WES) with a variant [c.899G>A p.(Gly300Asp)] with similar manifestations in his sibling and maternal aunt. 

## Case presentation

**Figure 1:**
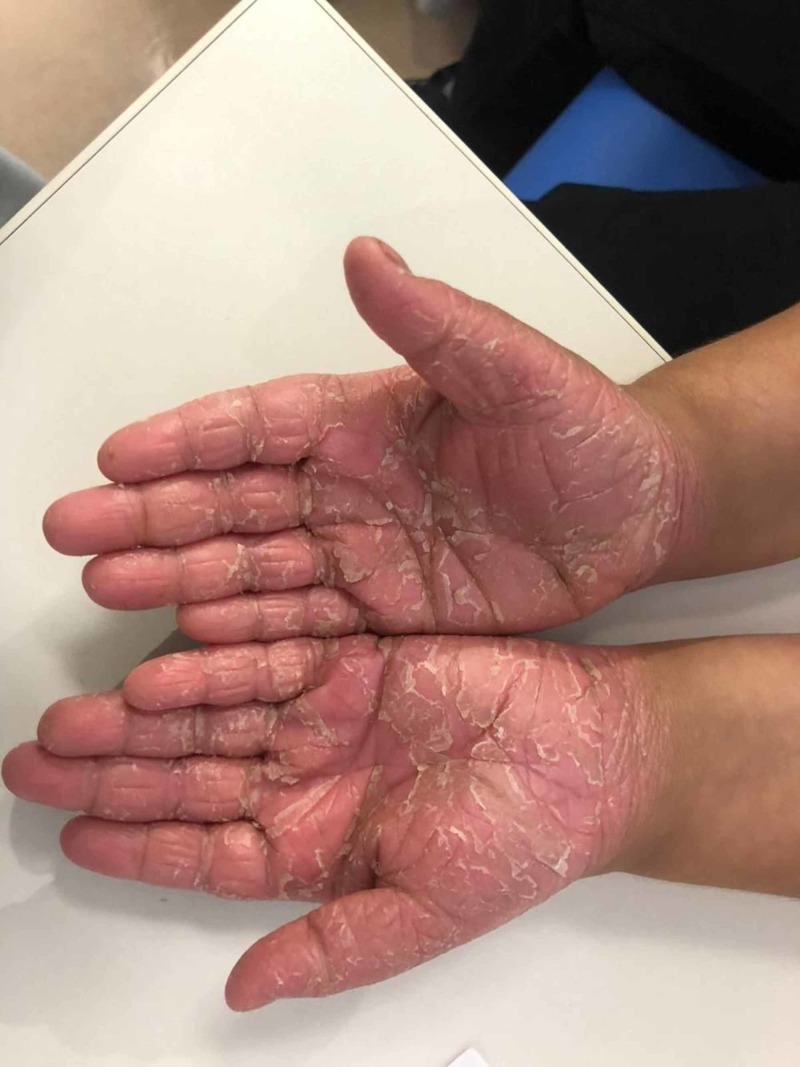
Erythematous hyperkeratotic skin plaques and papules with scales over the palmar aspect of both hands.

**Figure 2:**
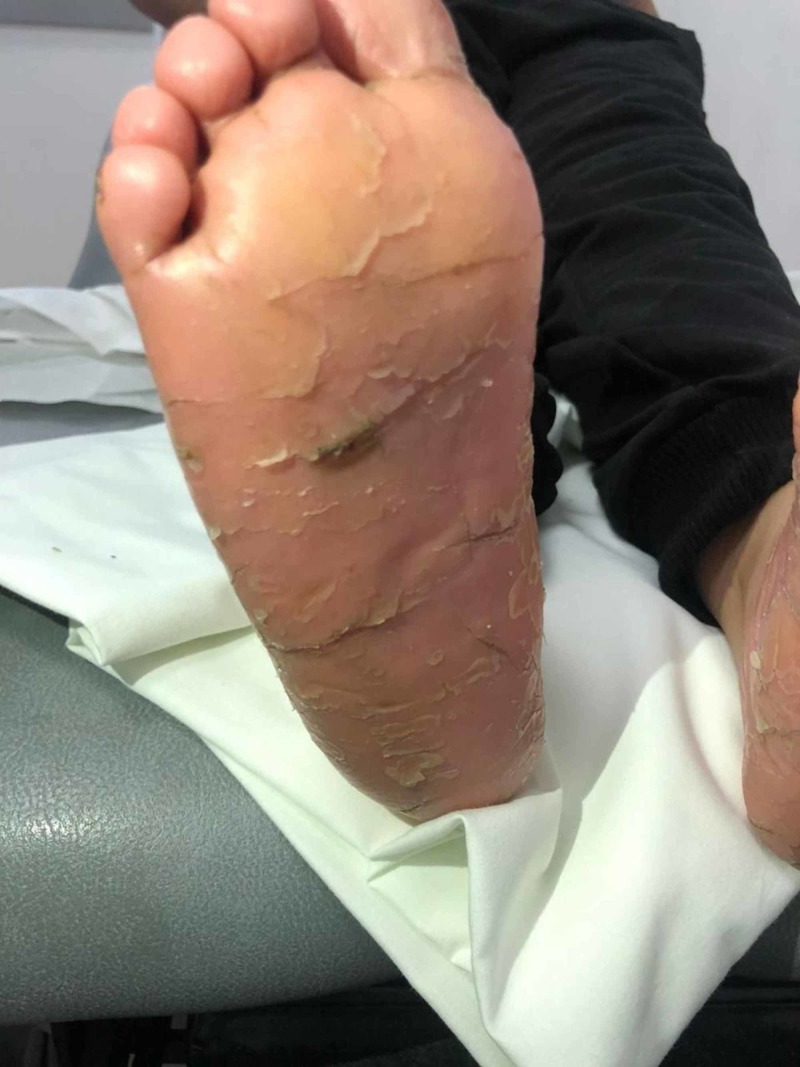
Erythematous hyperkeratotic skin plaques and papules with scales over the soles of both feet.

**Figure 3:**
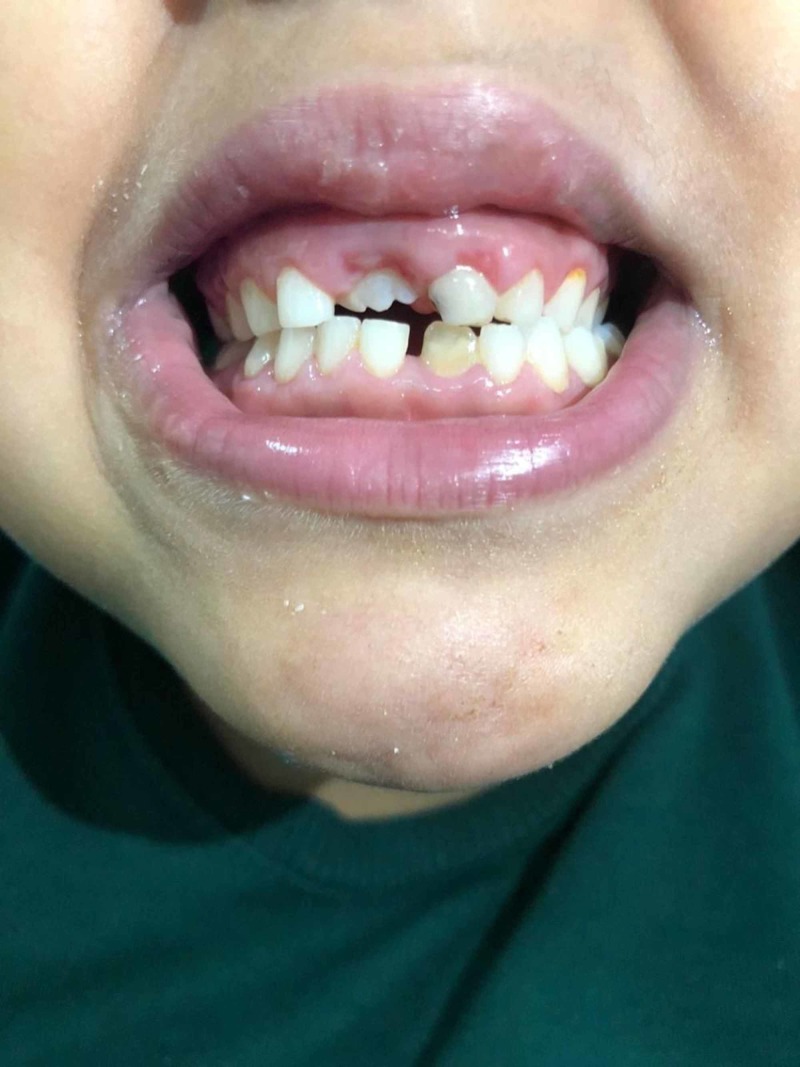
Oral gingiva showing periodontitis, missing teeth, and teeth with dental carries.

This is a seven-year-old boy who was born by spontaneous vaginal delivery at full term with a birth weight of 2.5 kg with no neonatal ICU admission. He first presented to the dermatology clinic at the National Guard Hospital, Makkah, Saudi Arabia when he was five years old complaining of dry scaly patches on the skin. The patient’s medical history revealed that it started at the age of four months in the form of desquamation and erythema on the hand and feet, sparing the trunk, back, and face. The father noticed that he is mouth breathing and has nasal discharge for three months with congested nasal turbinates. He is developmentally up to age and has taken all his vaccines. He has allergies to fish. On examination, he was active, alert with no dysmorphic features, and vitally stable. His height was 106 cm and his weight was 16 kg. Upon inspection, he was found to have erythematous hyperkeratotic skin plaques and papules with scales over the planter and palmar aspect of both hands with similar lesions observed on both feet, legs, scalp, and ears with nail pitting (Figures 1-2). All routine labs were within normal limits. He was diagnosed as having psoriasis and was treated by calcipotriol cream and moisturizing cream. During the follow ups he was not improving so he was given acitretin capsules and a skin punch biopsy was ordered. He started to show improvement after that. The results showed he had mild psoriasiform spongiotic dermatitis with no evidence of psoriasis. Accordingly, the patient was referred to pediatric genetics for further investigations. He was also referred to dental services for dental caries and delayed teething (Figure 3), and to an otolaryngologist to rule out nasal polyps. He was found to have positive family history, where his one-year-old brother and maternal aunt had similar conditions. A WES was done and it identified pathognomic variants in CTSC that cause autosomal recessive PLS (PALS; OMIM: 245000) (variant: c.899G>A p.(Gly300Asp) chr11:88027667). The patient did not follow up on his appointments with the dental care and otolaryngologist

## Discussion

A multidisciplinary approach is required with PLS to improve the prognosis and quality of life, as it has multiple manifestations involving different systems. Dental services are needed for the treatment of periodontitis and the prevention of its complications. There have been several bacteria that are associated with periodontal disease of PLS. *Actinobacillus actinomycetemcomitans* is the main suspect and it has been seen that it has a major role in the pathogenesis and progression of the periodontal disease [5-6]. Detection of the pathogens, appropriate antibiotic treatment, and extraction of the severely affected teeth can increase the viability of the healthy teeth. Moreover, oral retinoids recently have been used to promote the healthy growth of the teeth [6-7].

A dermatologist is involved for the skin manifestations that usually affect the hands and feet, whereas our patient is also affected in his legs, scalp, and ears. Emollients, salicylic acid, and topical steroids are used. Furthermore, retinoids are also used to treat the skin manifestations as it is thought that it can stimulate the humoral and cellular immunity. It has been found that with age there are improvements of the cutaneous lesions [8]. A cohort study of 47 patients has found that there is no significant correlation between the severity of the hyperkeratosis and periodontal destruction. However, a significant correlation has been found between the hands and feet, even though it was more severe in the feet [9]. Genetic counseling is recommended to confirm the diagnosis and to inform the family of the inheritance pattern of the disease as consanguinity is a significant risk factor for autosomal recessive disorders. Additionally, psychosocial support is recommended as PLS can affect the psychological, social, and esthetics aspects of children.

Multiple disorders have similar genetic and clinical features as PLS, such as Haim-Munk syndrome (HMS) (also known as “Cochin Jewish disorder” or “palmoplantar keratoderma with periodontitis and arachnodactyly and acroosteolysis”) [10]. It also has dermatological and periodontal manifestations, but also presents with pes planus, arachnodactyly, and acroosteolysis. Moreover, palmoplantar ectodermal dysplasia (PPED) may also mimic PLS as it has eight types that can include dermatological phenotypes, but has an autosomal dominant inheritance pattern [11].

## Conclusions

Papillon-Lefèvre syndrome is characterized by hyperkeratoderma over the palms and soles (palmoplantar hyperkeratosis) and generalized aggressive periodontitis. It is an autosomal recessive genetic disorder that is caused by a mutation in the gene that encodes CTSC. Moreover, PLS can easily be confused with psoriasis, unless confirmed by genetic testing. Early detection and proper management by a multidisciplinary team is essential to improve the quality of patients' life and better the outcomes. 

## References

[1] Papillon MM, Lefèvre P (1924). Deux cas de kératodermie palmaire et plantaire symétrique familiale (maladie de Meleda) chez le frère et la sœur. Coexistence dans les deux cas d'altérations dentaires graves. Bulletin de la Société Française de Dermatologie et de Vénéréologie.

[2] Dhanrajani PJ (2009). Papillon-Lefèvre syndrome: clinical presentation and a brief review. Oral Surg Oral Med Oral Pathol Oral Radiol Endodontol.

[3] Dalgic B, Bukulmez A, Sari S (2011). Eponym: Papillon-Lefèvre syndrome. Eur J Pediatrics.

[4] Acun Kaya F, Seyfioglu Polat Z, Akuzum Baran E, Tekin GG (2008). Papillon-Lefèvre syndrome. 3 years follow up: a case report. Int Dent Med Disord.

[5] Stabholz A, Taichman N, Soskolne W (1995). Occurrence of Actinobacillus actinomycetemcomitans and anti-leukotoxin antibodies in some members of an extended family affected by Papillon-Lefèvre syndrome. J Periodontol.

[6] Tinanoff N, Tempro P, Maderazo E (1995). Dental treatment of Papillon-Lefevre syndrome: 15-year follow-up. J Clin Periodontol.

[7] Gelmetti C, Nazzaro V, Cerri D, Fracasso L (1989). Long-term preservation of permanent teeth in a patient with Papillon-Lefevre syndrome treated with etretinate. Pediatric Dermatol.

[8] Lundgren T, Crossner C, Twetman S, Ullbro C (1996). Systemic retinoid medication and periodontal health in patients with Papillon-Lefevre syndrome. J Clin Periodontol.

[9] Ullbro C, Crossner C, Nederfors T, Alfadley A, Thestrup-Pedersen K (2003). Dermatologic and oral findings in a cohort of 47 patients with Papillon-Lefèvre syndrome. J Am Acad Dermatol.

[10] Pahwa P, Lamba A, Faraz F, Tandon S (2010). Haim-Munk syndrome. J Indian Soc Periodontol.

[11] Itin P (2014). Etiology and pathogenesis of ectodermal dysplasias. Am J Med Genet Part A.

